# Arthroscopic Repair of Acute Bony Bankart Lesion Following a Shoulder Dislocation Using Traction Sutures and Anchors: A Case Description

**DOI:** 10.7759/cureus.39811

**Published:** 2023-05-31

**Authors:** Mohd Nizlan Mohd Nasir, Paisal Hussin, Raymond Yeak Dieu Kiat, Johan Abdul Kahar

**Affiliations:** 1 Sports Surgery Division, Universiti Putra Malaysia, Serdang, MYS; 2 Orthopaedics, Columbia Asia Hospital, Puchong, MYS

**Keywords:** shoulder fracture dislocation, shoulder trauma, anchor suture repair, arthroscopic surgery, acute bony bankart

## Abstract

Management of shoulder dislocation can be challenging especially when glenoid bone fracture is involved. Bony Bankart lesion can be managed either through an open surgery or, of late, using arthroscopic technique. Arthroscopic bony Bankart repair is technically difficult, requiring specialized instruments to penetrate the bone fragment within the detached labrum. This case report describes an alternative way of doing an arthroscopic reattachment of an acute bony Bankart lesion using traction sutures, an accessory anteromedial portal and utilization of knotless anchors. A 44-year-old male technician was climbing a ladder when he slipped and fell directly on his left shoulder. Imaging revealed bony Bankart fracture with presence of ipsilateral greater tuberosity (GT) fracture and a Hill-Sachs lesion. In a right lateral position, arthroscopic reduction of the bony fragment was performed utilizing a Fibrewire® (Arthrex, Inc., Naples, FL, USA) suture as traction apparatus while securing the upper and lower tissue enveloping the bony Bankart fragment. An accessory portal was made lower down anteriorly to de-rotate the fragment, holding it in place while securing two Pushlock® (Arthrex, Inc.) anchors to the native glenoid. We then performed GT fixation using two cannulated screws. Check radiographs revealed acceptable reduction of the Bankart fragment. With careful case selection, arthroscopic repair of acute bony Bankart lesions is possible using special arthroscopic reduction maneuver and fixation technique with subsequent good outcome.

## Introduction

Traumatic shoulder dislocation often leads to separation of the anteroinferior labrum and capsule, known as a Bankart lesion. In 23% of cases, this separation is accompanied by bony avulsion of the glenoid rim, known as bony Bankart lesion [1]. Identification of this lesion is critical to facilitate proper preparation to reconstruct the glenoid rim during Bankart repair surgery. Traditionally, these lesions were dealt with by open bony reattachment along with capsular repair with good outcome [2]. Essentially, open repair is still performed for bony Bankart with 15% involvement [3]. Advancement in arthroscopic instrumentation and technique have allowed these lesions to be treated via arthroscopic reattachment [4-6]. However, certain techniques require specialized instruments [5] to penetrate the bone fragment for fixation stability. We present this case to share our experience in managing acute bony Bankart lesion and highlight our technique in reducing the bone fragment with standard arthroscopic instruments available in most trauma centers, along with some technical pearls in performing the procedure to ensure secure fixation of the bone fragment to the native glenoid rim.

## Case presentation

**Figure 1:**
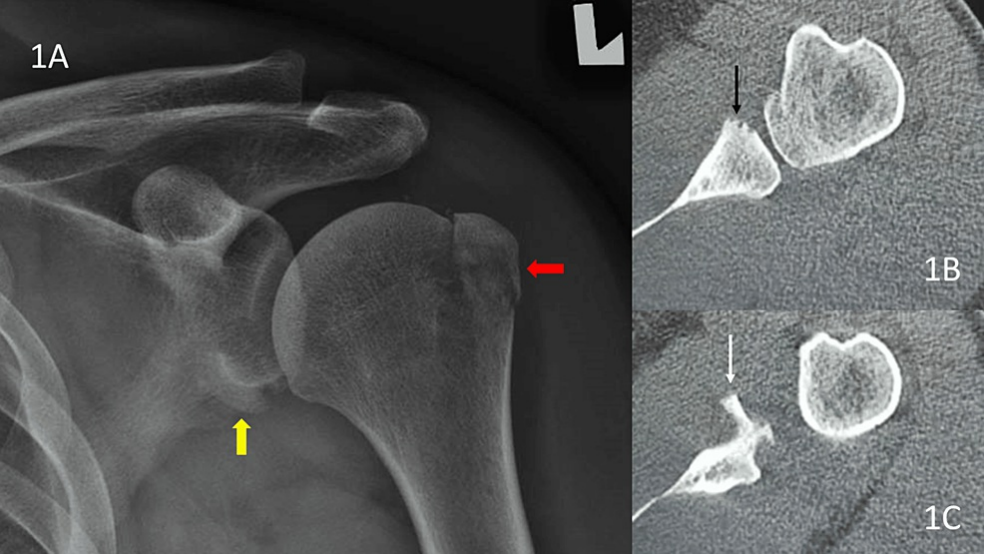
Plain anteroposterior (AP) radiograph and axial computed tomography (CT) images of the left shoulder. The greater tuberosity (GT) avulsion (red block arrow) with suspicious shadow at the glenoid neck (yellow block arrow) and the subsequent axial CT images showing antero-inferior glenoid defect (1B: black thin arrow) and a bony Bankart lesion displaced inferiorly (1C: white thin arrow).

A 44-year-old male technician was climbing a ladder when he slipped and fell directly on his left shoulder. He immediately felt pain in his left shoulder and was unable to move it. He was seen at the emergency department where plain radiographs showed an avulsion fracture of the greater tuberosity without any dislocation of the shoulder. No clear glenoid fracture was noted on plain radiograph. However, there was a suspicious shadow at the glenoid neck with disrupted glenoid outline, so a computed tomography (CT) scan was done, revealing a displaced bony Bankart lesion with a small Hill-Sachs lesion (Figure 1).

Based on history, physical examination and imaging findings, diagnoses of acute shoulder dislocation with spontaneous reduction, bony Bankart lesion and greater tuberosity (GT) avulsion fracture of the left shoulder were made. He consented to an arthroscopic repair on the bony Bankart lesion and screw fixation of the GT avulsion.

**Figure 2:**
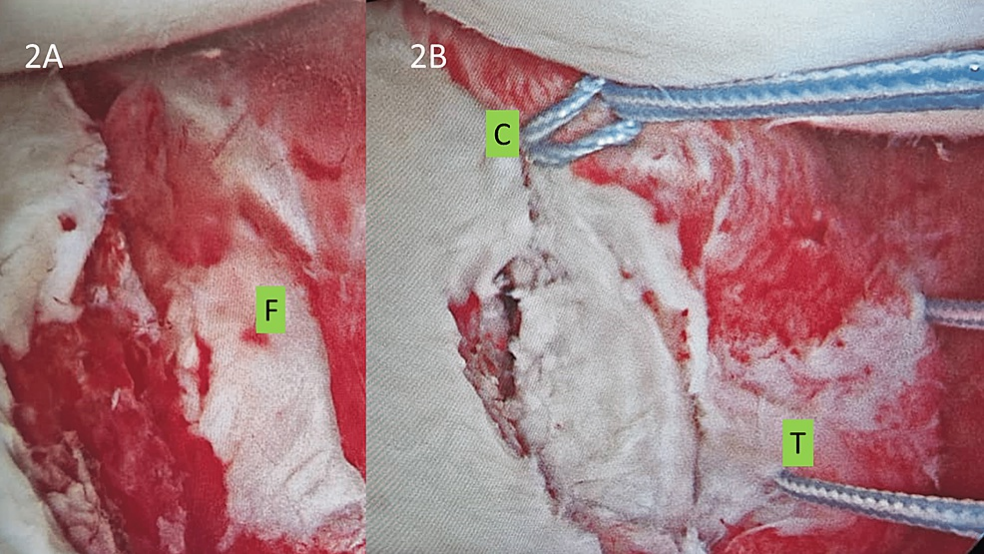
Fragment identification, reduction and suture fixation. The Bankart fragment (2A: F) was identified, debrided of any debris, tagged with a traction suture (2B: T), released until reduction was acceptable, and then secured with two cinched Fibrewires® above and below (2B: C) the attachment of the fragment to the labrum and capsule.

Pre-operatively the patient was put in a right lateral decubitus position with the affected arm on lateral traction - this is the preferred operative position by the main author for his arthroscopic Bankart repair (soft tissue or bony). Using standard posterior viewing and anterolateral portals, the bony Bankart fragment’s mobility and reducibility was initially assessed. Viewing was then switched to an anterosuperior portal, created within the rotator interval posterior to the long head of biceps tendon and just anterior to the leading edge of the supraspinatus tendon, for better visualization of the anteroinferior glenoid area and to facilitate reduction of the inferiorly-displaced Bankart fragment. Blood clots or chondral debris were removed, and the glenoid fracture edge was debrided. A tissue liberator (a tool commonly used during Bankart repair surgery to free any adhered labral tissue to the glenoid bone) was then introduced through the anterolateral portal and the fragment, along with its attached labral tissue and capsule was then mobilized and released so that the fragment can be reduced to the lateral edge of the glenoid surface with minimal tension. This reduction was aided by a traction suture (Fibrewire® [Arthrex, Inc., Naples, FL, USA] or equivalent high-tensile sutures) placed at the superior junction of the glenoid fragment and its attached labrum. Once satisfactory reduction has been achieved, two more Fibrewire® sutures were passed and cinched to the upper and lower tissue enveloping the bony Bankart fragment (Figure 2).

**Figure 3:**
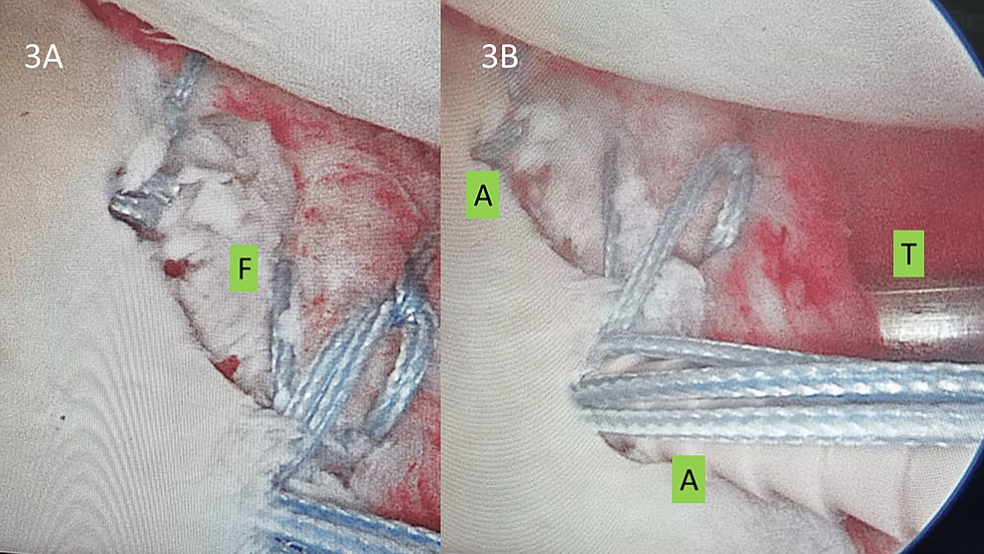
Fragment fixation with knotless anchors. The Bankart fragment (3A: F) was held in place by the Wissinger rod inserted through the 5 o’clock portal (3B: T). Two knotless Pushlock® anchors were then inserted to fix both the upper and lower sutures holding and securing the fragment in place (3B: A).

Even though there was a tendency for the fragment to rotate as the fixation points were limited to two ends of the fragment, this was overcome by the usage of a reduction tool (called switching stick or Wissinger rod, used commonly to aid the insertion of an arthroscopic cannula). An accessory portal was made lower down anteriorly (5 o’clock portal) and this rod was used to de-rotate the fragment while securing two Pushlock® anchors (Arthrex, Inc.) to the native glenoid, thus fixing the fragment securely in place. Once the anchors were deployed, the fragment remained stable (Figure 3). The Hill-Sachs lesion was left alone as measurements done pre-operatively and assessment done arthroscopically revealed a small lesion that will not subsequently affect stability of the shoulder.

**Figure 4:**
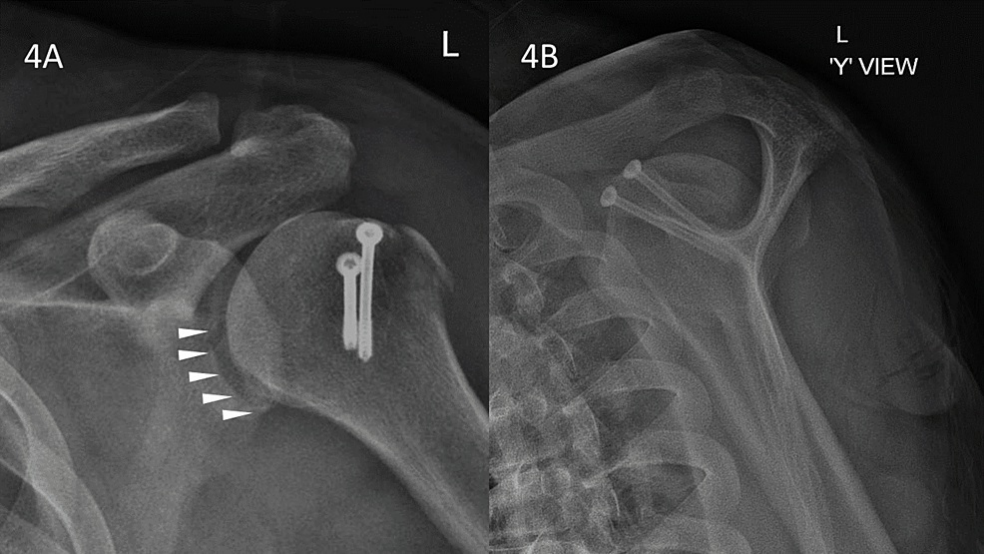
Check radiographs of the left shoulder. Plain radiographs (4A: anteroposterior and 4B: 'Y' view) showing the reduced greater tuberosity (GT) avulsion and the Bankart fragment secured in place with acceptable reduction (4A: white arrowheads).

Washout was then performed, and portals were closed in the usual manner. We then performed a mini-open GT fixation using two cannulated screws. Check radiographs showed a reduced and stable fragment fixation (Figure 4).

Post-operatively the patient was put in a neutral-wedge shoulder brace for six weeks, after which pendulum exercises were initiated. Active-assisted exercises were initiated after eight weeks, followed by active strengthening exercises after 12 weeks.

## Discussion

The aim of this demanding surgery is to reduce the Bankart fragment with no or minimal tension followed by fixing the fragment to the glenoid using suture anchors or screws. The desired result would be union of the fragment to the native glenoid to maintain shoulder stability. There are various techniques described in the literature for arthroscopic bony Bankart repair, with some authors utilizing specialized instruments [5,7] while others suggested double-row anchor configuration [5-7]. Cadaveric and biomechanical studies found superior strength with the usage of double-row anchor fixation [8,9].

Sugaya et al. [5] utilized a special suture passer strong enough to penetrate through the Bankart fragment and reported a good outcome with their series, however their 42 shoulders comprised chronic bony Bankart lesion with instability. Kuptniratsaikul et al. [7] added another anchor at the native glenoid medial to the reduced Bankart fragment and incorporated this anchor to their surface anchors to achieve a uniform compression across the fragment increasing its potential for healing and union. This fixation concept was initially described by Millet et al. [6].

We believe a simple two-point fixation above and below a Bankart fragment is good enough to achieve adequate compression, provided the following indications are strictly adhered to and these surgical steps are followed - (1) Acute bony Bankart lesion less than two weeks duration. Chronic fractures tend to have rounded edges due to resorption [10], making stable approximation of the fragment difficult and impossible to maintain. In chronic cases, the fragment also tends to displace medially, making release and reduction challenging. (2) No comminution of the fragment - hence it is possible to approximate the edges while holding the reduction stable by using a minimum of two fixation anchors. (3) Adequate visualization of the Bankart fragment through the anterosuperior portal. Proper debridement of the edge of the fragment is crucial. (4) Adequate release of the fragment from the glenoid neck and then pulling the fragment towards the glenoid surface using a grasper or a traction suture in place, as described above. (5) Close and tight capture on the soft tissue (labrum and capsule) of the fragment by the anchor-sutures - making subsequent anchor fixation strong enough to withstand any shoulder movements while the fragment unites. Cinching of the sutures to the soft tissue will make the fixation of the fragment stronger. (6) The use of an accessory portal and reduction tool such as the switching stick to hold the fragment in place while the anchors are being deployed. (7) Careful protection of the fixation during the post-operative period using a neutral-wedge brace for six weeks while union takes place.

## Conclusions

Acute bony Bankart repair is possible using arthroscopic reduction and fixation technique highlighted in this case report, with a good outcome. With careful case selection and adherence to appropriate indications, good healing of the Bankart fragment is expected, provided careful steps and precautions are taken during the surgery and during the post-operative period.
